# Differential expression of CK20, β-catenin, and MUC2/5AC/6 in Lynch syndrome and familial colorectal cancer type X

**DOI:** 10.1186/s12907-017-0052-1

**Published:** 2017-08-17

**Authors:** Stefan Haraldsson, Louise Klarskov, Mef Nilbert, Inge Bernstein, Jesper Bonde, Susanne Holck

**Affiliations:** 1 Department of Gastroenterology, Copenhagen University Hospital, Kettegaard Alle 29, DK-2650 Hvidovre, Denmark; 2 Department of Pathology, Herlev-Gentofte Hospital, Herlev, Denmark; 3 Clinical Research Centre, HNPCC register, Copenhagen University Hospital, Hvidovre, Denmark; 4 Institute of Clinical Sciences, Division of Oncology, Lund University, Lund, Sweden; 5 HNPCC register, Copenhagen University Hospital, Hvidovre, Denmark; 6 Department of Surgical Gastroenterology, Aalborg University Hospital, Aalborg, Denmark; 7 Department of Pathology and Clinical Research Center, Copenhagen University Hospital, Hvidovre, Denmark; 8 Department of Pathology, Copenhagen University Hospital, Hvidovre, Denmark

**Keywords:** Hereditary non-polyposis colorectal cancer, Fcctx, Lynch syndrome, Immunohistochemical profile

## Abstract

**Background:**

Hereditary non-polyposis colorectal cancer comprises Lynch syndrome and familial colorectal cancer type X (FCCTX). Differences in genetics, demographics and histopathology have been extensively studied. The purpose of this study is to characterize their immunoprofile of markers other than MMR proteins.

**Methods:**

We compared the expression patterns of cytokeratins (CK7 and CK20), mucins (MUC2/5 AC/6), CDX2 and β-catenin in Lynch syndrome and FCCTX.

**Results:**

Differences were identified for CK20 and nuclear β-catenin, which were significantly more often expressed in FCCTX than in Lynch syndrome (*p* < 0.001), whereas MUC2, MUC5AC and MUC6 were overexpressed in Lynch syndrome tumors compared with FCCTX tumors (*p* = 0.001, < 0.01, and < 0.001, respectively). We observed no differences in the expression patterns of CK7 and CDX2.

**Conclusions:**

In summary, we identified significant differences in the immunoprofiles of colorectal cancers linked to FCCTX and Lynch syndrome with a more sporadic-like profile in the former group and a more distinct profile with frequent MUC6 positivity in the latter group.

## Background

Identification of hereditary colorectal cancer provides an unprecedented possibility for cancer prevention through inclusion of family members at increased risk into surveillance programs. Identification and diagnostics of hereditary colorectal cancer requires joint efforts from clinicians, pathologists and geneticists. Hereditary non-polyposis colorectal cancer (HNPCC) represents the most common subset of hereditary colorectal cancer and comprises the major subsets Lynch syndrome and familial colorectal cancer type X (FCCTX). Germline mutations in one of the mismatch repair (MMR) genes and resultant microsatellite instability (MSI) characterize Lynch syndrome, whereas retained MMR function and unknown genetic causes characterize FCCTX [1–3]. Lynch syndrome shows a lower mean age at onset, an abundance of right-sided colon tumors and more frequent extracolonic tumors, whereas FCCTX is predominantly characterized by tumors in the distal colon and the rectum and shows a somewhat higher mean age at onset. Histologic differences include a “pushing” growth pattern, lymphocytic reactions, poor differentiation with mucinous and medullary growth patterns in Lynch syndrome and an infiltrative growth pattern, tumor budding, “dirty” necrosis, glandular differentiation and frequent node positivity in FCCTX [4–6].

The purpose of this study is to record the immunoprofile of markers well-described in colorectal carcinoma in general but, hitherto, incompletely studied in hereditary colorectal carcinomas. These include cytokeratins, mucin glycoproteins, and CDX2. Specifically, its discriminatory utility in FCCTX- vs Lynch syndrome cases is addressed, as is the feasibility of identifying FCCTX among colorectal carcinomas in general. Additionally, β-catenin is included, to compare the extent of the wnt pathway activation in the two hereditary cohorts, as Wnt-signaling genes are shown to be upregulated in FCCTX tumors.

## Methods

### Patient identification and accrual of samples

Patients were identified through the national Danish HNPCC register (http://www.hnpcc.dk). In Denmark, patients with suspected or verified hereditary colorectal cancer are reported to this register by laboratories and responsible clinicians. In Denmark, colorectal cancer diagnostics includes reflex testing for MMR protein expression using antibodies against MLH1, PSM2, MSH2 and MSH6. Cases with loss of expression are, if applicable, and implying the patient provides consent, referred to genetic counselling. Genetic counselling is performed by clinical genetic counsellors and clinical geneticists at 4 departments countrywide. Following genetic diagnostics, Lynch syndrome was defined as presence of disease-predisposing MMR gene variants (classes 4 and 5) and FCCTX was defined as families that fulfilled the Amsterdam criteria, but had tumors with retained MMR function and for the majority of families also genetic MMR gene testing without mutations. The histopathological profiles of the 2 cohorts have been presented in Klarskov et al. [4]. In total, 65 colorectal cancers from 60 individuals in 41 FCCTX families and 68 Lynch syndrome tumors from 62 individuals in 41 families were studied including 2 synchronous and 3 metachronous tumor pairs. Hematoxylin & eosin stained slides from the formalin fixed-paraffin embedded (FFPE) tissue samples selected were reviewed to ensure representation of the deep tumor margin [7]. Clinical data were collected from the pathology reports and tumor location was classified as proximal or distal in relation to the splenic flexure. Tumor differentiation was classified as poorly differentiated/undifferentiated or highly/moderately differentiated.

The study was granted ethical permission by the Region Hovedstaden ethical review board (H-D-2007-032).

### Immunohistochemical staining

Immunohistochemical stainings were performed on fresh 4-μm sections from FFPE tissue that was deparaffinized in Tissue clear. Antigen retrieval was achieved by PT-Link and 3-in-1 buffer, pH 9 (Dako). The sections were processed in a Dako autostainer (Dako, Denmark), applying the antibodies targeting CK7/20, MUC2/5 AC/6, CDX2, and β-catenin (Table 1). The Envision Detection Kit (DakoCytomation) was used according to the manufacturer’s instructions and tissue sections were counterstained with Meyer’s hematoxylin, dehydrated, mounted on coated slides, and dried 1 h at 60°. The immunostainings were scored semiquantitatively by two independent pathologists (LK, SH), blinded to mutational status. A 5-tier scale was applied, using the following categories; no staining, < 5%, 5–50%, 51–95% and > 95% stained tumor cells. In the analyses, the stainings were dichotomized into negative (< 5% staining) and positive (≥ 5% staining). For CDX2 only strong expression, equivalent to the intensity of the normal mucosa was considered, for the other immunostainings, labelling intensity was not considered. Where interpretative doubts arose and in case of diverse readings, which rarely exceeded one category, consensus was reached by conference.

**Table 1:** Antibodies

Antibody	Clone	Dilution	Manufacturer
CK7	OV-TL 12/30	RTU	Dako, DK
CK20	KS20.8	RTU	Dako, DK
MUC2	Ccp58	1:25	Novocastra/Leica, UK
MUC5Ac	CLH2	1:200	Novocastra/Leica, UK
MUC6	CLH5	1:50	Novocastra/Leica, UK
β-catenin	β-catenin 1	RTU	Dako, DK
Cdx2	Dak-CDX2	RTU	Dako, DK

*RTU* ready to use

### Statistics

All data were entered in duplicate in Epidata and exported to SPSS 17.0 for statistical analysis. Statistical differences between the groups were determined using Pearson’s χ^2^-test for categorical and independent samples, t-test for continuous parametric data. *P*-values less than 0.05 were considered statistically significant.

## Results

### Clinical data

Clinical data are summarized in Table 2. Significant differences applied as regards age (younger mean age in Lynch syndrome, *p* < 0.001), tumor location (78% of FCCTX tumors were left-sided versus 26% of Lynch syndrome, *p* < 0.001) and extent of differentiation (54% of Lynch syndrome poorly differentiated/undifferentiated versus 17% of FCCTX tumors (*p* < 0.001)).

**Table 2:** Demographics and tumor differentiation

Variables	FCCTX (*n* = 65)	Lynch (*n* = 68)	*P* value
Median age, (range), years	60 (28–83)	52 (25–82)	< 0.001
Gender, male	34 (52%)	28 (41%)	NS
Tumor site^a^			< 0.001
Right	13 (20%)	48 (71%)	
Left	51 (78%)	18 (26%)	
Not indicated	1 (2%)	2 (3%)	
Histological differentiation			< 0.001
High/moderate	54 (83%)	31 (56%)	
Poor/undifferentiated	11 (17%)	37 (54%)	

*NS* not significant

^a^Cut-off: splenic flexure

### Immunohistochemistry

**Fig. 1:**
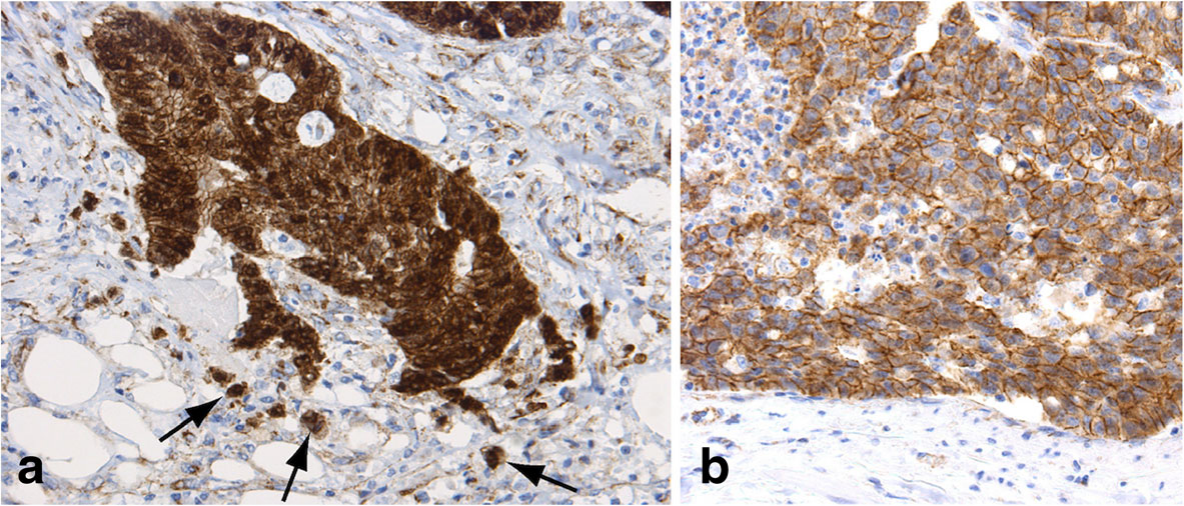
β-catenin expression in a FCCTX carcinoma (**a**) and in a Lynch syndrome carcinoma (**b**): The invasive front of FCCTX carcinoma (**a**) with prominent nuclear labelling of the single, budding tumor cells (some are arrowed) and in most tumor cells sited in the more coherent group. This aberrant profile characterized 52% of the FCCTX cohort, but only 16% of the Lynch syndrome tumors. Note additionally the infiltrative quality of the invasive front of the tumor, another feature of FCCTX tumors [4]. The invasive front of a Lynch syndrome carcinoma (**b**) with normal staining pattern, i.e. labelling confined to the tumor cell membranes, specifically absence of nuclear labelling. Note the pushing quality of the invasive border (below) and absence of budding cells, additional features of Lynch syndrome carcinomas [4]

**Fig. 2:**
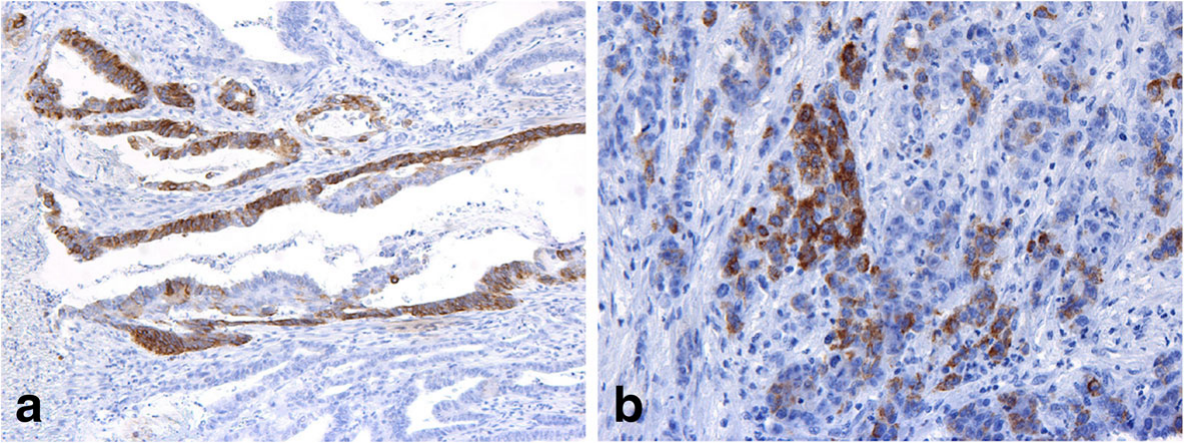
Focal MUC6 expression in two LS carcinomas (**a** and **b**): 25% of the Lynch syndrome carcinomas were focally positive. This profile was noted in highly/moderately differentiated examples (prominent glandular component) (**a**), as well as in poorly differentiated/undifferentiated cases (absence of glandular elements) (**b**). Merely 3% of the FCCTX cohort displayed MUC6 expression

**Fig. 3:**
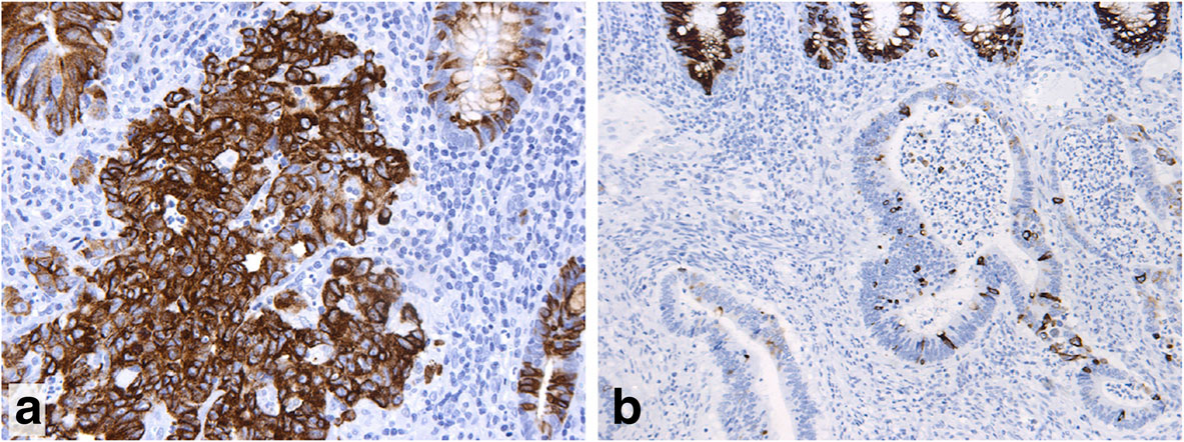
MUC2 expression in a Lynch syndrome carcinoma (**a**) and in a FCCTX carcinoma (**b**): 88% of the Lynch syndrome carcinomas were MUC2 positive, compared to 65% of the FCCTX cases. The Lynch syndrome carcinoma illustrated in A, is extensively decorated (portions of non-neoplastic crypts appear to the right). The FCCTX carcinoma, illustrated in B, comprises only few scattered immunopositive cells. Top of the field displays basis of several non-neoplastic crypts

The immunohistochemical profiles for five of the seven markers studied significantly differed between colorectal cancers linked to FCCTX and Lynch syndrome (Tables 3 and 4). Aberrant, nuclear staining for β-catenin (Fig. 1a) was more common in FCCTX tumors, whereas the β-catenin staining more often was normal, i.e. confined to the cell membranes (Fig. 1b), in Lynch syndrome (*p* < 0.001). Compared to FCCTX tumors, Lynch syndrome tumors displayed significantly more often expression of the tested MUC glycoproteins. The difference in MUC expression was particularly prominent for MUC6 (Fig. 2) (*p* < 0.001), less so for MUC2 (Fig. 3) (*p* = 0.001), and MUC5AC (*p* < 0.01). FCCTX tumors showed more frequent CK20 expression than did Lynch syndrome tumors (*p* < 0.001) with an equal distribution in right and left side of the large bowel. In Lynch syndrome tumors, CK20 expression patterns correlated to tumor location with more frequent expression of CK20 in right-sided tumors (77%) than in the left-sided tumors (50%) (*p* = 0.03).

**Table 3:** Immunoprofiles of FCCTX and Lynch syndrome-associated CRC

Marker	FCCTX (*n* = 65)	Lynch (*n* = 68)	*P* value
CK20, n (%)	62 (95)	46 (68)	< 0.001
CK7, n (%)	8 (12)	10 (15)	NS
MUC2, n (%)	42 (65)	60 (88)	0.001
MUC5AC, n (%)	7 (11)	20 (29)	< 0.01
MUC6, n (%)	2 (3)	17 (25)	< 0.001
CDX2, n (%)	64 (99)	63 (93)	NS
β-catenin, nuclear, n (%)	34 (52)	11 (16)	< 0.001

*NS* not significant

**Table 4:** Combined CK7/CK20-profiles of FCCTX and Lynch syndrome-associated CRC

Combination	FCCTX (*n* = 65)	Lynch (*n* = 68)	*P* value
CK7−/CK20+, n (%)	54 (83)	43 (63)	0.01
CK7+/CK20+, n (%)	8 (12)	3 (4)	NS
CK7−/CK20-, n (%)	3 (5)	15 (22)	0.003
CK7+/CK20-, n (%)	0 (0)	7 (10)	0.008

*NS* not significant

The CK7 expression rate was 12 and 15% in FCCTX and Lynch syndrome, respectively.

The combined CK7/20 patterns showed significant differences between Lynch syndrome tumors and FCCTX tumors (Table 4). The prevailing CK7−/CK20+ profile was identified in 83% of the FCCTX cases and in 63% of the Lynch syndrome tumors. CK7+/CK20+ profile was the second most common combination (12%) in FCCTXs, but was rare (4%) in Lynch syndrome tumors, which more often (22%) showed a CK7−/CK20- profile.

Expression of CDX2 was abundant without statistical significant differences between Lynch syndrome and FCCTX (93 and 99%, respectively).

## Discussion

Immunohistochemical staining is commonly applied as adjunct diagnostics in colorectal cancer. To this end profiles for the expression of cytokeratins, CDX2 and mucin glycoproteins are well-established markers [8–11] that are available in colorectal cancer diagnostics in most pathology laboratories. Since application of such profiles may be relevant also in hereditary cancer diagnostics, our aim was to define these profiles in the two major HNPCC subsets of colorectal cancer. Moreover, immunohistochemical profiling may contribute to the molecular understanding of these subsets. Herein, a large fraction of FCCTX tumors has been shown to harbour *APC* (adenomatous polyposis coli) mutations [12], which motivated evaluation of β-catenin staining as a candidate marker.

Tumors linked to Lynch syndrome and FCCTX showed significant differences, primarily related to frequent expression of CK20 and nuclear β-catenin in FCCTX and relative over-expression of MUC2, MUC5AC and MUC6 in Lynch syndrome. Sánchez-Tomé et al. likewise reported differences in the immunoprofile of FCCTX carcinomas (27 cases) – and Lynch syndrome carcinomas (18 cases) based on markers selected to analyze colorectal carcinogenesis, including SMAD4, COX2, MUC1, and P53 [13]. Despite differences in the selected immunopanels in these two studies the differences between the two hereditary cohorts in both studies are remarkable, and suggest that these profiles may be of clinical diagnostic relevance. We further found that FCCTX tumors generally mimicked the profile of the non-neoplastic colorectal mucosa, with CK20+, MUC5AC- and MUC6-, which contrasted to the expression pattern in the Lynch syndrome tumors.

Frequent (70–100%) expression of CK20 has been reported in unselected colorectal cancers [8, 14–20]. The reduced expression of CK20 observed in Lynch syndrome is in line with reduced CK20 levels in MSI tumors [9] and in poorly differentiated tumors (54% of Lynch syndrome carcinomas vs 17% in the FCCTX cases in the present material were poorly differentiated). In this context, it is noteworthy that cytokeratin filaments are relatively stable during transformation to carcinoma [21], a quality that is lost in a proportion of the Lynch syndrome carcinomas. CK20 expression has also been reported to correlate with the anatomical location with more abundant expression in the distal colon [17, 22]. This distribution pattern was, however, not observed in the current cohorts, which showed no side differences in FCCTX and higher expression in proximal than in distal Lynch syndrome tumors.

CK7 expression did not differ between Lynch syndrome tumors and FCCTX tumors and paralleled the expression levels (10–22%) in unselected and sporadic colorectal cancers reported in the literature [8, 15, 17].

The prevailing CK7−/CK20+ cytokeratin profile is reported in 55–77% of colorectal cancer in general [9] and was also the predominant profile in the hereditary subsets, though more frequent in FCCTX compared to Lynch syndrome. The second most common cytokeratin profile in unselected tumors is the CK7+/CK20+ combination identified in 15% of tumors [8, 9, 23]. This pattern was also the second leading profile in the FCCTX cases. In Lynch syndrome tumors the CK7−/CK20- profile was the second most common pattern, conceivably reflecting a higher frequency of poorly differentiated tumors [4, 24], speculatively a result of the hypermutated state of MSI tumors. Of further note is the CK7+/20- combination in 10% of our Lynch syndrome tumors. Bayrak et al. [8] reported this rare pattern in only 2% of unselected cases, whereas this profile specifically was noted in high grade, right-sided colorectal cancers, properties suggestive of Lynch syndrome. According to our results, this unusual profile seems to exclude FCCTX and might suggest Lynch syndrome. Additionally, the CK7−/20- combination makes FCCTX unlikely. The other combinations of CK7 and CK20 lack, however, discriminatory value.

Loss of expression of CDX2 in colorectal cancer has been reported as a negative prognostic marker [25]. CDX2 expression in the HNPCC-associated colorectal cancers in the present study was high, which is in accordance with the relatively good prognosis characterizing these cancers, and did not differ from some series on colorectal carcinoma in general [23, 26–28]. Diversities in study design, specifically use of tissue micro array, can readily contribute to the lower values noted in other reports of colorectal cancers [15, 29, 30].

Our study on the secreted gel-forming mucins (MUC2, MUC5AC, and MUC6) demonstrated significantly higher values in the Lynch syndrome than in the FCCTX tumors. In this context, the idea that the MSI status may influence mucus production, by altering the genes involved [31] is noteworthy. Mucinous differentiation, though it may not reach the 50% required for classification of a mucinous tumor, is frequent in Lynch syndrome tumors. Of note is the observation that MUC expression levels identified in Lynch syndrome tumors were higher than described in reported series of mucinous tumors [32–34]. MUC6 expression has been suggested to inhibit tumor invasion in pancreatic cancer [35], which may apply to colorectal cancer as well and could play a role in the favourable prognosis known to characterize Lynch syndrome tumors. Indeed, a recent report on the clinical significance of secreted gel-forming MUCs in colorectal carcinomas demonstrated a favorable influence on the outcome in case of gain in aberrant MUC expression, particularly of MUC6 expression [36]. The MUC profile in the FCCTX subset was more akin to that of unselected colorectal cancers with MUC2 expression reported in 40–54%, MUC5AC in 6–10% and MUC6 in 4% [17, 37].

Nuclear translocation of β-catenin is a marker of dysregulated Wnt signalling. Diverse mechanisms may induce this event in colorectal carcinoma, the major cause being dysfunction of the *APC* gene [38]. In total, 52% of the present FCCTX tumors showed aberrant nuclear β-catenin, which is in line with unselected and MMR-proficient series [26, 39]. The findings also roughly correlate with those of Franscisco et al. [12] who reported *APC* mutation in 62% of their FCCTX cases. Other MMR-mutation negative, familial series (including 20, 24, and 44 cases) have reported lower frequencies of nuclear β-catenin [40–42]. Given the presumed heterogeneity of FCCTX tumors, partial inclusion of MSI tumors, differences in study design and limited-size series disparities can be anticipated [12]. *β-catenin* mutation, another cause of aberrant β-catenin, probably contributes to the occasional aberrant β-catenin expression in Lynch syndrome tumors. Based on the current immunohistochemical study, nuclear β-catenin expression in Lynch syndrome tumors is uncommon compared to its prevalence in FCCTX (16% vs. 52%). In concert, nuclear β-catenin expression was previously recorded in merely 19% of colorectal cancer from 118 Lynch syndrome patients in a study conducted by some of us [43]. Further markers of the wnt-signalling pathway may be of interest in future studies of FCCTX tumors.

Distinct gene expression patterns have been demonstrated in colorectal cancers linked to Lynch syndrome and FCCTX and overall support that FCCTX tumors mimic sporadic MMR-proficient tumors [44, 45]. These data and the immunohistochemical expression differences we describe herein suggest that evaluation of key markers should be exploited for future diagnostic application. FCCTX tumors are characterized by chromosomal instability and deregulation of genes and proteins involved in e.g. chromosomal segregation, genomic stability, apoptosis, proliferation, growth inhibition, angiogenesis and migration [44]. The limited data available point to involvement of pathways related to G protein-coupled signaling, proliferation and migration. In line with this, FCCTX tumors frequently show infiltrative growth patterns and presence of dirty necrosis [45]. Lynch syndrome tumors show frequent deregulation of genes involved in the cell cycle progression and in the oxidative phosphorylation pathway as well as immune response genes. Regarding the latter, studies are currently exploring the role of immunohistochemical evaluation of specific immune checkpoint proteins in the context of immunotherapy in colorectal cancer. The role for DNA methylation changes remains to be defined, though the gene-specific methylation of MLH1 is a hallmark of the hypermutable phenotype in sporadic MSI tumors and global hypomethylation has been demonstrated in FCCTX and has been shown to interfere with chromosomal instability.

The strengths of the present study include clinically well-defined and relatively large study populations, immunohistochemical evaluations on whole sections in contraposition to the limited areas available by tissue micro arrays (the latter a potential source of error, as previously pinpointed [23]), and evaluation by two pathologists who were blinded to patient data. The study design allows for descriptive analyses only, which is a limitation and data on somatic mutations of KRAS, NRAS and BRAF are not included.

## Conclusions

Significant differences in the immunohistochemical profiles of colorectal cancers linked to Lynch syndrome and FCCTX are not restricted to MMR-proteins. In particular, CK20, MUC2, MUC5AC, MUC6 and β-catenin showed disparate expression patterns that may in part be ascribed to clinicopathologic factors such as tumor location, mucinous components, differentiation, and MSI-status, conceivably reflecting diverse underlying genetic mechanism(s). The chosen antibody panel did not allow differentiation between FCCTX and colorectal carcinoma in general. As knowledge on FCCTX genetic(s) emerges, translation into novel biomarkers, useful in discriminating FCCTX from its sporadic counterpart can be anticipated.

## Abbreviations

APC: Adenomatous polyposis coli
CDX: Caudal type homeobox
CK: Cytokeratin
FCCTX: Familial Colorectal Cancer Type X
FFPE: Formalin fixed paraffin embedded
HNPCC: Hereditary non-polyposis colorectal cancer
MMR: Mismatch repair
MSI: Microsatellite instability
MUC: Mucin
